# Supplementary material for: DNA-based identification of plants and the genomic nature of plant species differences
Source: Commun Biol. 2026 Mar 26;9:673. doi: 10.1038/s42003-026-09858-7 (PMC13183946; doi:10.1038/s42003-026-09858-7)
Supplement: Supplementary file 2 — Supporting Information [file 42003_2026_9858_MOESM2_ESM.pdf]

Supporting Information for

DNA-based Identification of Plants and the Genomic Nature of Plant Species Differences

Wu Huang[1,2,6,*], De-Zhu Li[3,4,5], Alexandre Antonelli[6,7,8,9], Christine D. Bacon[7], Lian-Ming Gao[3,4,5], Catherine Kidner[1,10], R. Toby Pennington[1,11], Douglas E. Soltis[12,13], Pamela Soltis[12,13], Jeannine Cavender-Bares[14], Camille Christe[15], Kyle G. Dexter[1,16,17], Yanqian Ding[1,2], Mario Durán-Castillo[2], Mario Fernández-Mazuecos[18], Elliot M. Gardner[19], Morgan R. Gostel[20], Margarita Hernandez[21], Andrew L. Hipp[22], Johanna R. Jantzen[12,13], Jacob B. Landis[23], Xiu-Qun Liu[24], Andre A. Naranjo[25], James Nicholls[1,26], Han-Tao Qin[3,4], Jeffrey P. Rose[27], Nicolas Salamin[28], Rowan Schley[11], Philipp M. Schlüter[29], Jessica D. Stephens[30], Matthew A. Streisfeld[31], Natascha D. Wagner[32], Xiao-Quan Wang[33], Qiu-Yun Jenny Xiang[34], Alex D. Twyford[1,2,*], Peter M. Hollingsworth[1,*]

* Wu Huang, Alex D. Twyford, Peter M. Hollingsworth

Email: whuang@rbge.org.uk, Alex.Twyford@ed.ac.uk, phollingsworth@rbge.org.uk

This PDF file includes:

Figures S1 to S5
Supplementary References

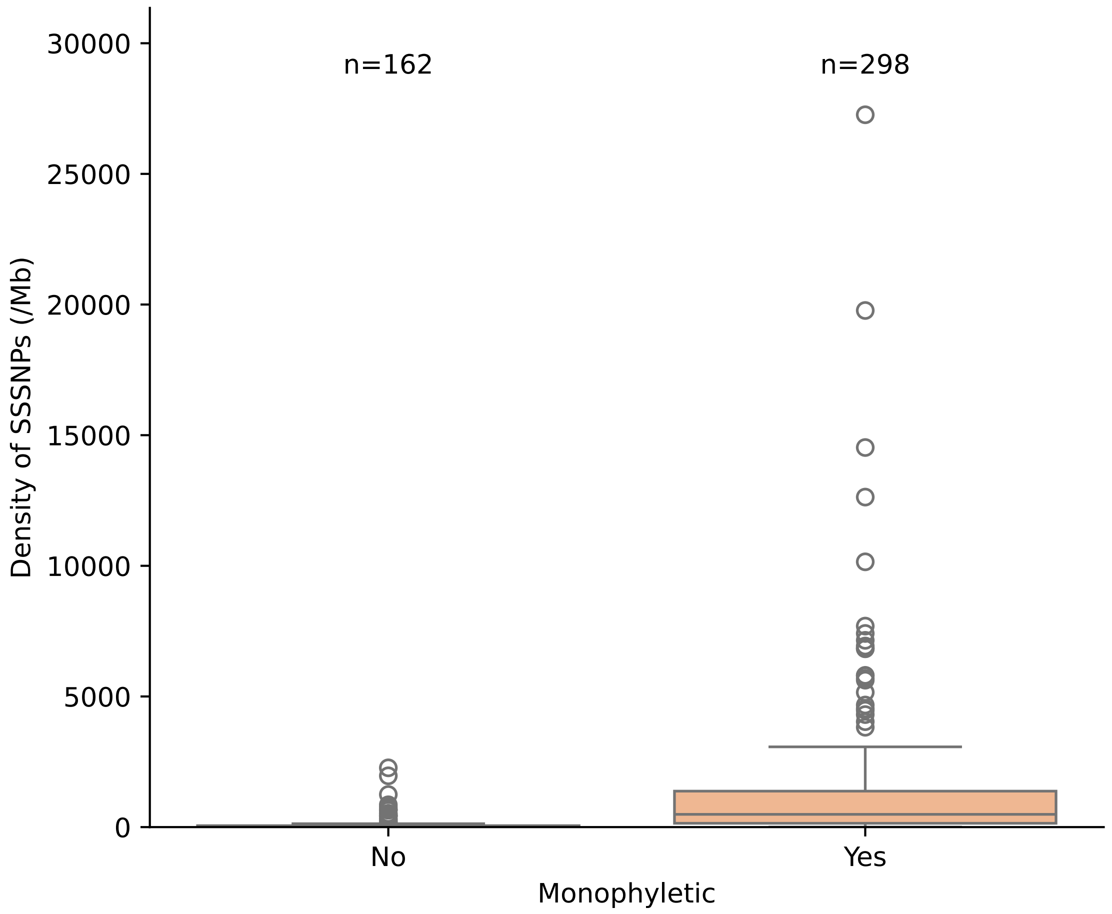

Fig. S1. The density of SSSNPs comparison between monophyletic species and non-monophyletic species (all genera) (p-value <0.001).

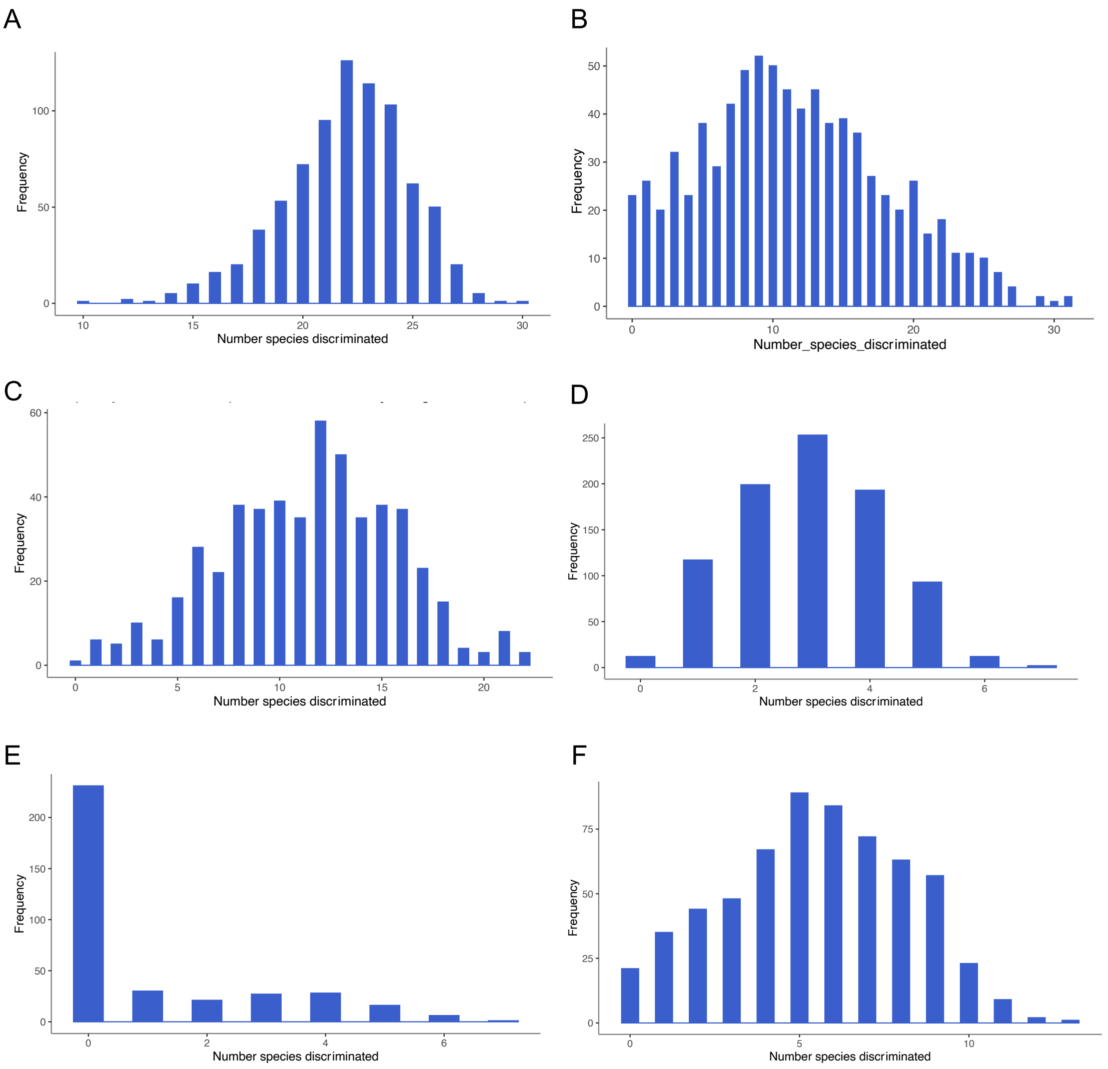

Fig. S2. Frequency distribution of the number of species discriminated by individual loci. The x-axis is ordered from the genes with the lowest discriminatory power to those with the greatest discriminatory power. A. *Geonoma*, B. *Inga*, C. *Artocarpus*, D. *Tsuga*, E. *Polemonium*, and F. *Capurodendron*.

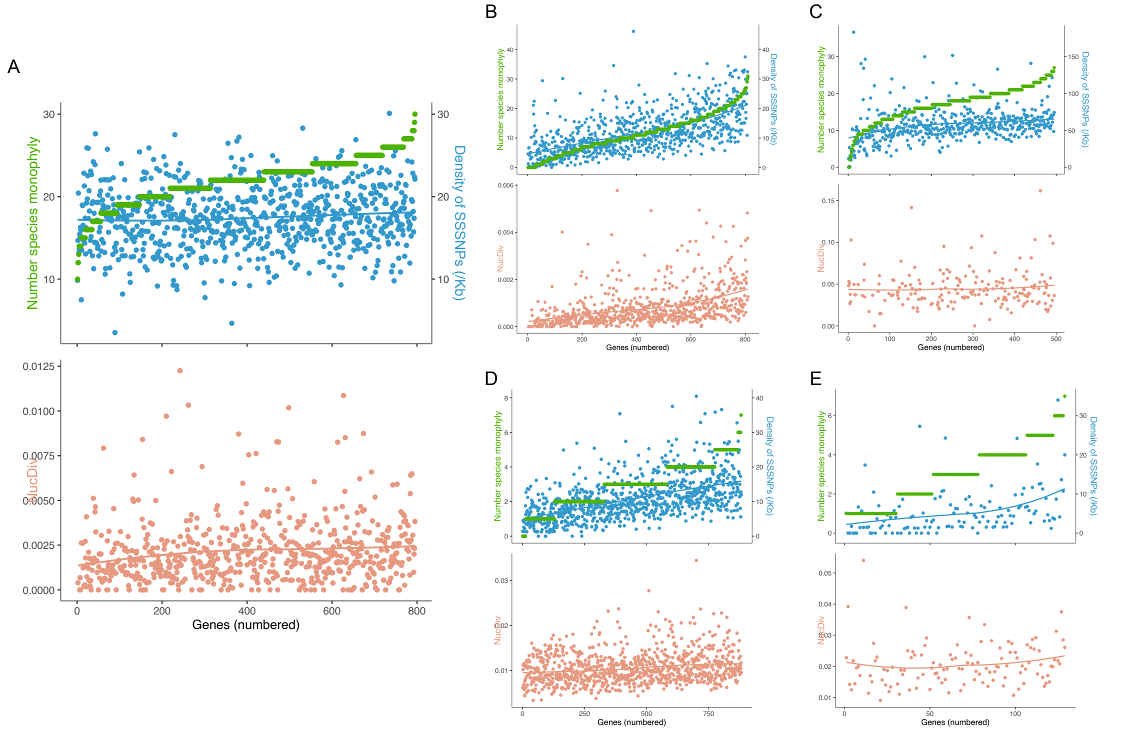

Fig. S3. The genetic features of individual genes for five genera – A. *Geonoma*, B. *Inga*, C. *Artocarpus*, D. *Tsuga*, and E. *Polemonium*. The x-axis is ordered by individual loci from the worst to the best-performing genes in terms of the number of species they discriminate. The green dots represent the number of species resolved as monophyletic by each locus; the blue dots represent the density of SSSNPs (per kilobases) for each locus, and the peach dots represent the nucleotide diversity of a given locus across the whole genus.

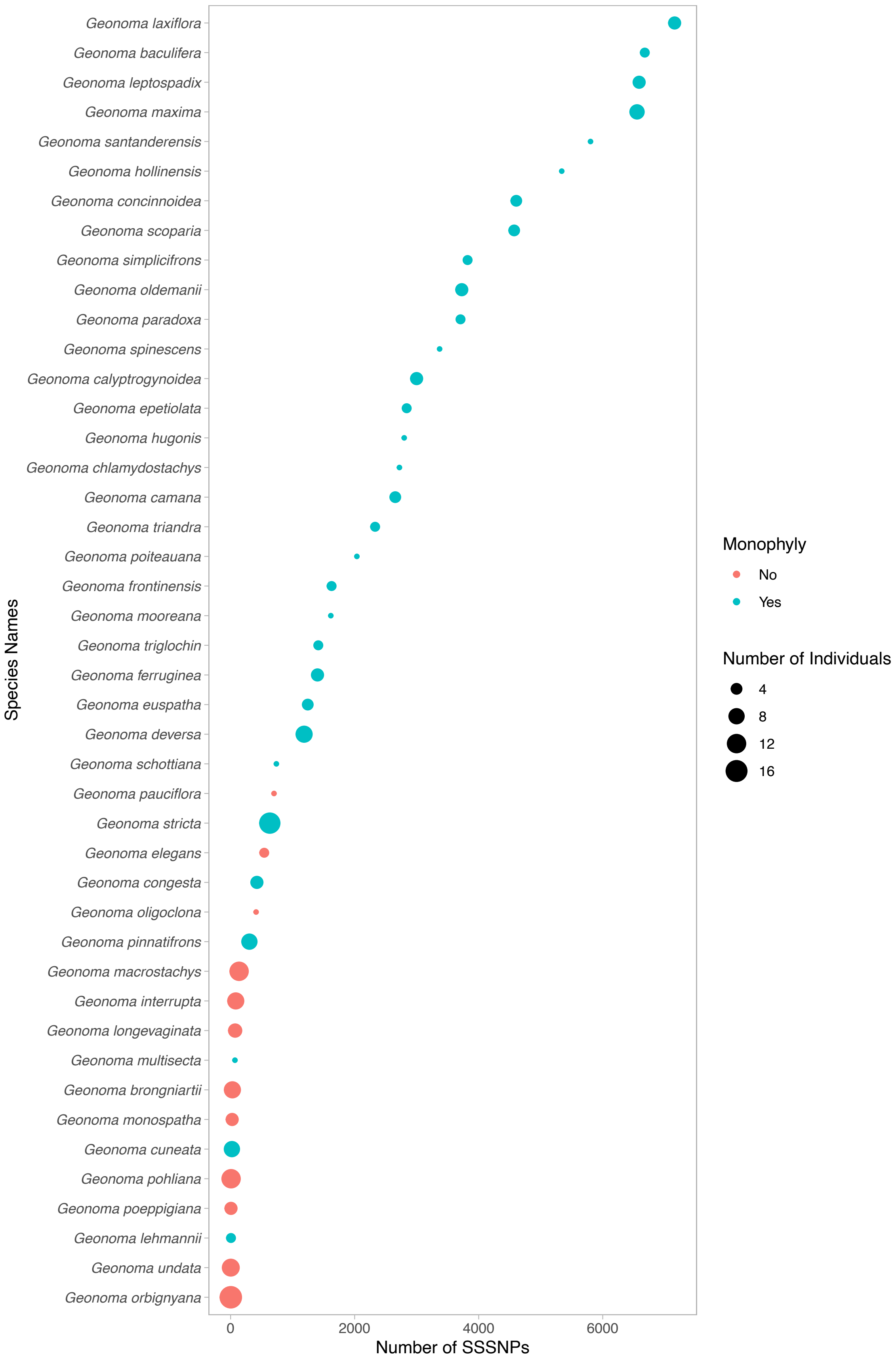
Geonoma laxiflora
Geonoma baculifera
Geonoma leptospadix
Geonoma maxima
Geonoma santanderensis
Geonoma hollinensis
Geonoma concinnoidea
Geonoma scoparia
Geonoma simplicifrons
Geonoma oldemanii
Geonoma paradoxa
Geonoma spinescens
Geonoma calyptrogynoidea
Geonoma epetiolata
Geonoma hugonis
Geonoma chlamydostachys
Geonoma camana
Geonoma triandra
Geonoma poiteauana
Geonoma frontinensis
Geonoma mooreana
Geonoma triglochin
Geonoma ferruginea
Geonoma euspatha
Geonoma deversa
Geonoma schottiana
Geonoma pauciflora
Geonoma stricta
Geonoma elegans
Geonoma congesta
Geonoma oligoclona
Geonoma pinnatifrons
Geonoma macrostachys
Geonoma interrupta
Geonoma longevaginata
Geonoma multisecta
Geonoma brongniartii
Geonoma monospatha
Geonoma cuneata
Geonoma pohliana
Geonoma poeppigiana
Geonoma lehmannii
Geonoma undata
Geonoma orbignyana
Species Names
0
2000
4000
6000
Number of SSSNPs
Monophyly
No
Yes
Number of Individuals
4
8
12
16

Fig. S4. The distribution of species-specific SNPs for all multi-sampled plant species of genus *Geonoma* (Figures for all other genera can be found in the supplementary folder Fig.S4. Number_of_SSSNPs_vs_Monophyly_correlation_for_all_genus which can found at can be found at Zenodo DOI: 10.5281/zenodo.17603347). The blue dots represent a species being monophyletic, and the peach dots represent non-monophyletic; the size of the dots represents the number of individuals sampled in each species.

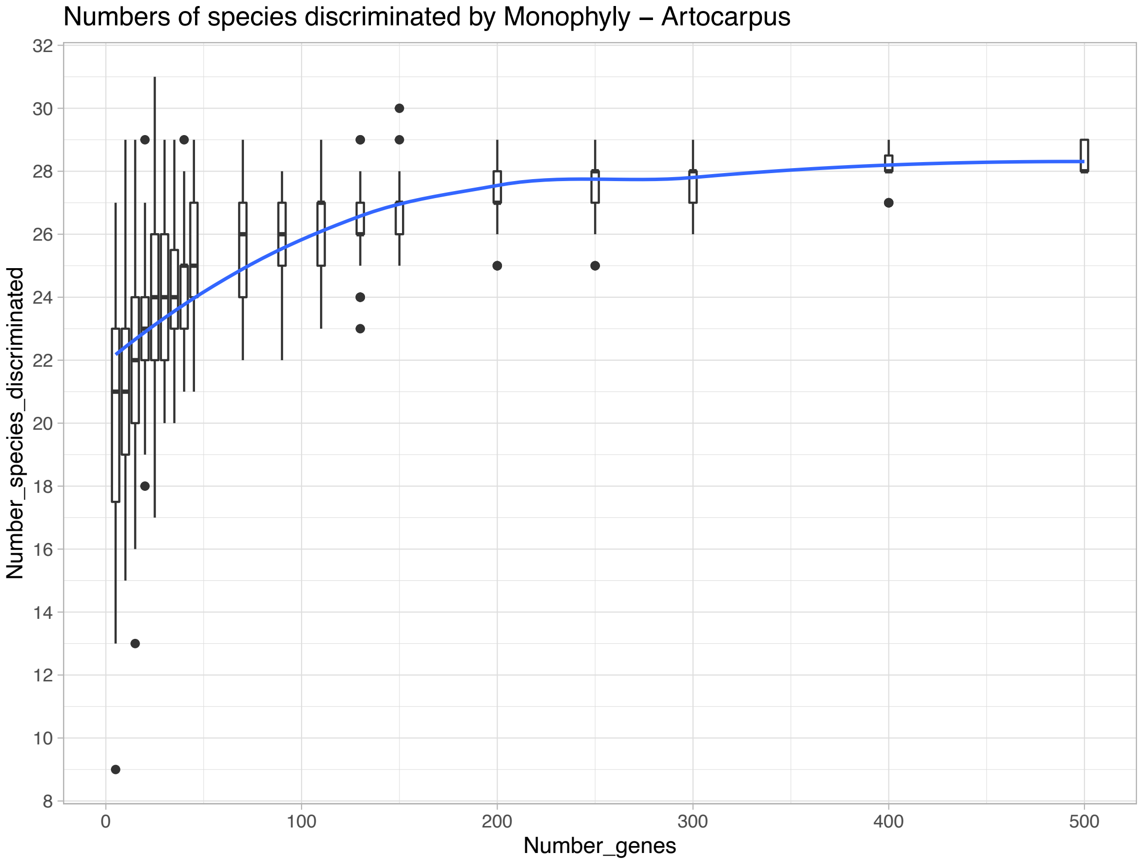

Fig. S5. The subsampling curve of the individual genera by SNPs of genus *Autocarpus*. The name of each individual dataset is on the top of each plot. Figures for all other genera could be found in the supplementary folder Fig.S5. Downsampling – boxplot which can found at can be found at Zenodo DOI: 10.5281/zenodo.17603347.

Supplementary References

1. P. Huhn *et al.*, How challenging RADseq data turned out to favor coalescent-based species tree inference. A case study in *Aichryson* (Crassulaceae). *Mol. Phylogenet. Evol.* **167**, 107342 (2022).
2. S. Dong *et al.*, Nuclear loci developed from multiple transcriptomes yield high resolution in phylogeny of scaly tree ferns (Cyatheaceae) from China and Vietnam. *Mol. Phylogenet. Evol.* **139**, 106567 (2019).
3. M. G. Stetter, K. J. Schmid, Analysis of phylogenetic relationships and genome size evolution of the *Amaranthus* genus using GBS indicates the ancestors of an ancient crop. *Mol. Phylogenet. Evol.* **109**, 80-92 (2017).
4. D. I. Ojeda *et al.*, Phylogenomic analyses reveal an exceptionally high number of evolutionary shifts in a florally diverse clade of African legumes. *Mol. Phylogenet. Evol.* **137**, 156-167 (2019).
5. M. Durán-Castillo, A. Hudson, Y. Wilson, D. L. Field, A. D. Twyford, A phylogeny of *Antirrhinum* reveals parallel evolution of alpine morphology. *New Phytol.* **233**, 1426-1439 (2022).
6. A. Otero, M. Fernandez-Mazuecos, P. Vargas, Evolution in the model genus *Antirrhinum* based on phylogenomics of topotypic material. *Front. Plant Sci.* **12**, 631178 (2021).
7. O. W. White, J. A. Reyes-Betancort, M. A. Chapman, M. A. Carine, Geographical isolation, habitat shifts and hybridisation in the diversification of the Macaronesian endemic genus *Argyranthemum* (Asteraceae). *New Phytol.* **228**, 1953-1971 (2020).
8. E. M. Gardner *et al.*, Paralogs and off-target sequences improve phylogenetic resolution in a densely-sampled study of the breadfruit genus (*Artocarpus*, Moraceae). *Syst. Biol.* 10.1093/sysbio/syaa073 (2020).
9. Y. Okuyama *et al.*, Radiation history of Asian *Asarum* (sect. *Heterotropa*, Aristolochiaceae) resolved using a phylogenomic approach based on double-digested RAD-seq data. *Ann. Bot.***126**, 245-260 (2020).
10. X. Wang *et al.*, Genome-wide RAD sequencing data provide unprecedented resolution of the phylogeny of temperate bamboos (Poaceae: Bambusoideae). *Sci. Rep.* **7**, 11546 (2017).
11. X. Q. Wang, L. Zhao, D. A. Eaton, D. Z. Li, Z. H. Guo, Identification of SNP markers for inferring phylogeny in temperate bamboos (Poaceae: Bambusoideae) using RAD sequencing. *Mol. Ecol. Resour.* **13**, 938-945 (2013).
12. R. Comito, I. Darbyshire, C. Kiel, L. McDade, A. E. Fisher, A RADseq phylogeny of *Barleria* (Acanthaceae) resolves fine-scale relationships. *Mol. Phylogenet. Evol.* **169**, 107428 (2022).
13. N. Wang, L. J. Kelly, H. A. McAllister, J. Zohren, R. J. A. Buggs, Resolving phylogeny and polyploid parentage using genus-wide genome-wide sequence data from birch trees. *Mol. Phylogenet. Evol.* **160**, 107126 (2021).
14. I. E. Jordon-Thaden *et al.*, A basic ddRADseq two-enzyme protocol performs well with herbarium and silica-dried tissues across four genera. *Applications in Plant Sciences* **8**, e11344 (2020).
15. R. J. Schley *et al.*, Introgression across evolutionary scales suggests reticulation contributes to Amazonian tree diversity. *Mol. Ecol.* 10.1111/mec.15616 (2020).
16. F. J. Nge, J. Kellermann, E. Biffin, M. Waycott, K. R. Thiele, Historical biogeography of *Pomaderris* (Rhamnaceae): Continental vicariance in Australia and repeated independent dispersals to New Zealand. *Mol. Phylogenet. Evol.* **158**, 107085 (2021).
17. J. R. Brock, A. A. Donmez, M. A. Beilstein, K. M. Olsen, Phylogenetics of Camelina Crantz. (Brassicaceae) and insights on the origin of gold-of-pleasure (*Camelina sativa*). *Mol. Phylogenet. Evol.* **127**, 834-842 (2018).
18. H. Yang *et al.*, Genetic divergence between *Camellia sinensis* and its wild relatives revealed via genome-wide SNPs from RAD sequencing. *PLoS One* **11**, e0151424 (2016).

19. S. Federman, M. J. Donoghue, D. C. Daly, D. A. R. Eaton, Reconciling species diversity in a tropical plant clade (*Canarium*, Burseraceae). *PLoS One* **13**, e0198882 (2018).
20. C. Christe, C. G. Boluda, D. Koubinova, L. Gautier, Y. Naciri, New genetic markers for Sapotaceae phylogenomics: More than 600 nuclear genes applicable from family to population levels. *Mol. Phylogenet. Evol.* **160**, 107123 (2021).
21. M. Slenker *et al.*, Allele sorting as a novel approach to resolving the origin of allotetraploids using Hyb-Seq data: a case study of the Balkan mountain endemic *Cardamine barbaraeoides*. *Front. Plant Sci.* **12**, 659275 (2021).
22. T. Villaverde *et al.*, Bridging the micro- and macroevolutionary levels in phylogenomics: Hyb-Seq solves relationships from populations to species and above. *New Phytol.* **220**, 636-650 (2018).
23. Y. Hou *et al.*, RAD-seq data point to a northern origin of the arctic-alpine genus *Cassiope* (Ericaceae). *Mol. Phylogenet. Evol.* **95**, 152-160 (2016).
24. E. L. Spriggs, M. E. Fertakos, Evolution of *Castanea* in North America: restriction-site-associated DNA sequencing and ecological modeling reveal a history of radiation, range shifts, and disease. *Am. J. Bot.* **108**, 1692-1704 (2021).
25. W. Zhou, A. J. Harris, Q. Y. Xiang, Phylogenomics and biogeography of *Torreya* (Taxaceae) – Integrating data from three organelle genomes, morphology, and fossils and a practical method for reducing missing data from RAD-seq. *JSE* 10.1111/jse.12838 (2022).
26. S. P. Kinosian, W. D. Pearse, P. G. Wolf, Cryptic diversity in the model fern genus *Ceratopteris* (Pteridaceae). *Mol. Phylogenet. Evol.* **152**, 106938 (2020).
27. J. R. Bombonato *et al.*, The potential of genome-wide RAD sequences for resolving rapid radiations: a case study in Cactaceae. *Mol. Phylogenet. Evol.* **151**, 106896 (2020).
28. L. A. Frost, N. O'Leary, L. P. Lagomarsino, D. C. Tank, R. G. Olmstead, Phylogeny, classification, and character evolution of tribe *Citharexyleae* (Verbenaceae). *Am. J. Bot.* **108**, 1982-2001 (2021).
29. P. Hamon *et al.*, Genotyping-by-sequencing provides the first well-resolved phylogeny for coffee (*Coffea*) and insights into the evolution of caffeine content in its species: GBS coffee phylogeny and the evolution of caffeine content. *Mol. Phylogenet. Evol.* **109**, 351-361 (2017).
30. J. C. Charr *et al.*, Complex evolutionary history of coffees revealed by full plastid genomes and 28,800 nuclear SNP analyses, with particular emphasis on *Coffea canephora* (Robusta coffee). *Mol. Phylogenet. Evol.* **151**, 106906 (2020).
31. M. R. Gostel, K. A. Coy, A. Weeks, Microfluidic PCR-based target enrichment: A case study in two rapid radiations of *Commiphora* (Burseraceae) from Madagascar. *JSE* **53**, 411-431 (2015).
32. K. Lindelof, J. A. Lindo, W. Zhou, X. Ji, Q. Y. Xiang, Phylogenomics, biogeography, and evolution of the blue- or white-fruited dogwoods (*Cornus*)—Insights into morphological and ecological niche divergence following intercontinental geographic isolation. *JSE* **58**, 604-645 (2020).
33. I. Larridon *et al.*, Tackling rapid radiations with targeted sequencing. *Front. Plant Sci.* **10**, 1655 (2019).
34. C. Arbizu, H. Ruess, D. Senalik, P. W. Simon, D. M. Spooner, Phylogenomics of the carrot genus (*Daucus*, Apiaceae). *Am. J. Bot.* **101**, 1666-1685 (2014).
35. F. Martínez-Flores *et al.*, Extended studies of interspecific relationships in *Daucus* (Apiaceae) using DNA sequences from ten nuclear orthologues. *Bot. J. Linn.* **191**, 164-187 (2019).
36. X. R. Ke *et al.*, Nuclear and plastid phylogenomic analyses provide insights into the reticulate evolution, species delimitation, and biogeography of the Sino-Japanese disjunctive *Diabelia* (Caprifoliaceae). *JSE* 10.1111/jse.12815 (2022).
37. Y. Hou *et al.*, Thousands of RAD-seq loci fully resolve the phylogeny of the highly disjunct arctic-alpine genus *Diapensia* (Diapensiaceae). *PLoS One* **10**, e0140175 (2015).

38. M. L. Gaynor *et al.*, Biogeography and ecological niche evolution in Diapensiaceae inferred from phylogenetic analysis. *JSE* **58**, 646-662 (2020).
39. M. Soto Gomez *et al.*, A customized nuclear target enrichment approach for developing a phylogenomic baseline for *Dioscorea* yams (Dioscoreaceae). *Applications in Plant Sciences* **7**, e11254 (2019).
40. A. G. Linan *et al.*, RAD-sequencing reveals patterns of diversification and hybridization, and the accumulation of reproductive isolation in a clade of partially sympatric, tropical island trees. *Mol. Ecol.* **30**, 4520-4537 (2021).
41. O. M. Vargas, E. M. Ortiz, B. B. Simpson, Conflicting phylogenomic signals reveal a pattern of reticulate evolution in a recent high-Andean diversification (Asteraceae: Astereae: *Diplostephium*). *New Phytol.* **214**, 1736-1750 (2017).
42. L. S. Xu, Y. S. Chen, Phylogeny, origin, and dispersal of *Dubyaea* (Asteraceae) based on Hyb-Seq data. *Mol. Phylogenet. Evol.* **164**, 107289 (2021).
43. D. M. White, M. B. Islam, R. J. Mason-Gamer, Phylogenetic inference in section *Archerythroxylum* informs taxonomy, biogeography, and the domestication of coca (*Erythroxylum* species). *Am. J. Bot.* **106**, 154-165 (2019).
44. H. Becher *et al.*, Maintenance of species differences in closely related tetraploid parasitic *Euphrasia* (Orobanchaceae) on an isolated island. *Plant Commun.* **1**, 100105 (2020).
45. Y. Wang *et al.*, Phylogenomics and rapid diversification of the genus *Eutrema* on the Qinghai–Tibet Plateau and adjacent regions. *JSE* 10.1111/jse.12828 (2022).
46. M. S. Dillenberger, J. W. Kadereit, Simultaneous speciation in the European high mountain flowering plant genus *Facchinia* (*Minuartia* s.l., Caryophyllaceae) revealed by genotyping-by-sequencing. *Mol. Phylogenet. Evol.* **112**, 23-35 (2017).
47. Z. C. Qi *et al.*, Phylogenomics of polyploid *Fothergilla* (Hamamelidaceae) by RAD-tag based GBS—insights into species origin and effects of software pipelines. *JSE* **53**, 432-447 (2015).
48. J. R. Starr *et al.*, Targeted sequencing supports morphology and embryo features in resolving the classification of Cyperaceae tribe Fuireneae s.l. *JSE* **59**, 809-832 (2021).
49. O. Loiseau *et al.*, Targeted capture of hundreds of nuclear genes unravels phylogenetic relationships of the diverse neotropical palm tribe Geonomateae. *Front. Plant Sci.* **10**, 864 (2019).
50. E. Y. Hwang *et al.*, Genetic diversity and phylogenetic relationships of annual and perennial *Glycine* species. *G3 (Bethesda)* **9**, 2325-2336 (2019).
51. R. M. Bateman, Phenotypic versus genotypic disparity in the Eurasian orchid genus *Gymnadenia*: exploring the limits of phylogeny reconstruction. *Syst. Biodivers.* **19**, 400-422 (2021).
52. S. Martin-Hernanz *et al.*, Maximize resolution or minimize error? using Genotyping-By-Sequencing to investigate the recent diversification of *Helianthemum* (Cistaceae). *Front. Plant Sci.* **10**, 1416 (2019).
53. J. D. Stephens, W. L. Rogers, C. M. Mason, L. A. Donovan, R. L. Malmberg, Species tree estimation of diploid *Helianthus* (Asteraceae) using target enrichment. *Am. J. Bot.* **102**, 910-920 (2015).
54. G. J. Baute, G. L. Owens, D. G. Bock, L. H. Rieseberg, Genome-wide genotyping-by-sequencing data provide a high-resolution view of wild *Helianthus* diversity, genetic structure, and interspecies gene flow. *Am. J. Bot.* **103**, 2170-2177 (2016).
55. S. K. Hirota *et al.*, Evolutionary history of *Hemerocallis* in Japan inferred from chloroplast and nuclear phylogenies and levels of interspecific gene flow. *Mol. Phylogenet. Evol.* **164**, 107264 (2021).
56. Z. Y. Du, A. J. Harris, Q. J. Xiang, Phylogenomics, co-evolution of ecological niche and morphology, and historical biogeography of buckeyes, horsechestnuts, and their relatives (Hippocastaneae, Sapindaceae) and the value of RAD-Seq for deep evolutionary inferences back to the Late Cretaceous. *Mol. Phylogenet. Evol.* **145**, 106726 (2020).

57. M. A. Gerschwitz-Eidt, J. W. Kadereit, Genotyping-by-sequencing (GBS), ITS and cpDNA phylogenies reveal the existence of a distinct Pyrenean/Cantabrian lineage in the European high mountain genus *Homogyne* (Asteraceae) and imply dual westward migration of the genus. *Alpine Botany* 10.1007/s00035-018-0212-7 (2018).
58. M. J. Yoo, B. Y. Lee, S. Kim, C. E. Lim, Phylogenomics with Hyb-Seq unravels Korean *Hosta* evolution. *Front. Plant Sci.* **12**, 645735 (2021).
59. A. Otero *et al.*, A snapshot of progenitor-derivative speciation in *Iberodes* (Boraginaceae). *Mol. Ecol.* 10.1111/mec.16459 (2022).
60. J. A. Nicholls *et al.*, Using targeted enrichment of nuclear genes to increase phylogenetic resolution in the neotropical rain forest genus *Inga* (Leguminosae: Mimosoideae). *Front. Plant Sci.* **6**, 710 (2015).
61. D. J. Gates, D. Pilson, S. D. Smith, Filtering of target sequence capture individuals facilitates species tree construction in the plant subtribe *Iochrominae* (Solanaceae). *Mol. Phylogenet. Evol.* **123**, 26-34 (2018).
62. P. Munoz-Rodriguez *et al.*, Discovery and characterization of sweetpotato's closest tetraploid relative. *New Phytol.* **234**, 1185-1194 (2022).
63. A. S. Zalmat, V. A. Sotola, C. C. Nice, N. H. Martin, Genetic structure in Louisiana *Iris* species reveals patterns of recent and historical admixture. *Am. J. Bot.* **108**, 2257-2268 (2021).
64. M. Ogishima *et al.*, Frequent chloroplast capture among *Isodon* (Lamiaceae) species in Japan revealed by phylogenies based on variation in chloroplast and nuclear DNA. *Plant Species Biol.* **34**, 127-137 (2019).
65. Q. Zhao *et al.*, Pan-genome analysis highlights the extent of genomic variation in cultivated and wild rice. *Nat. Genet.* **50**, 278-284 (2018).
66. X. Y. Mu *et al.*, Phylogeny and divergence time estimation of the walnut family (Juglandaceae) based on nuclear RAD-Seq and chloroplast genome data. *Mol. Phylogenet. Evol.* **147**, 106802 (2020).
67. X. Y. Mu, M. Sun, P. F. Yang, Q. W. Lin, Unveiling the identity of wenwan walnuts and phylogenetic relationships of Asian juglans species using restriction site-associated DNA-sequencing. *Front. Plant Sci.* **8**, 1708 (2017).
68. K. A. Uckele, R. P. Adams, A. E. Schwarzbach, T. L. Parchman, Genome-wide RAD sequencing resolves the evolutionary history of serrate leaf *Juniperus* and reveals discordance with chloroplast phylogeny. *Mol. Phylogenet. Evol.* **156**, 107022 (2021).
69. C. C. Howard, A. A. Crowl, T. S. Harvey, N. Cellinese, Peeling back the layers: First phylogenomic insights into the *Ledebouriinae* (Scilloideae, Asparagaceae). *Mol. Phylogenet. Evol.* **169**, 107430 (2022).
70. N. A. Tinker *et al.*, Classification and characterization of species within the genus *Lens* using Genotyping-by-Sequencing (GBS). *PLoS One* **10** (2015).
71. M. Liber, I. Duarte, A. T. Maia, H. R. Oliveira, The history of lentil (*Lens culinaris* subsp. *culinaris*) domestication and spread as revealedby Genotyping-by-Sequencing of wild and landrace accessions. *Front. Plant Sci.* **12**, 628439 (2021).
72. M. Fernandez-Mazuecos *et al.*, Resolving recent plant radiations: Power and robustness of Genotyping-by-Sequencing. *Syst. Biol.* **67**, 250-268 (2018).
73. J. Cohen, Phylogenomics, floral evolution, and biogeography of *Lithospermum* L. (Boraginaceae). *Mol. Phylogenet. Evol.* **166**, 107317 (2022).
74. M. Kandziora, P. Sklenar, F. Kolar, R. Schmickl, How to tackle phylogenetic discordance in recent and rapidly radiating groups? developing a workflow using *Loricaria* (Asteraceae) as an example. *Front. Plant Sci.* **12**, 765719 (2021).
75. N. Contreras-Ortiz, G. W. Atchison, C. E. Hughes, S. Madriñán, Convergent evolution of high elevation plant growth forms and geographically structured variation in Andean *Lupinus* (Fabaceae). *Bot. J. Linn.* **187**, 118-136 (2018).
76. M. M. Hanes, S. Shell, T. Shimu, C. Crist, S. Machkour-M'Rabet, The phylogeographic history of *Megistostegium* (Malvaceae) in the dry, spiny thickets of southwestern

Madagascar using RAD-seq data and ecological niche modeling. *Ecol. Evol.* **12**, e8632 (2022).
77. C. Paetzold, K. R. Wood, D. A. R. Eaton, W. L. Wagner, M. S. Appelhans, Phylogeny of Hawaiian *Melicope* (Rutaceae): RAD-seq resolves species relationships and reveals ancient introgression. *Front. Plant Sci.* **10**, 1074 (2019).
78. R. L. Stubbs *et al.*, A phylogenomic perspective on evolution and discordance in the Alpine-Arctic plant clade *Micranthes* (Saxifragaceae). *Front. Plant Sci.* **10**, 1773 (2019).
79. M. Curto, C. Schachtler, P. Puppo, H. Meimberg, Using a new RAD-sequencing approach to study the evolution of *Micromeria* in the Canary islands. *Mol. Phylogenet. Evol.* **119**, 160-169 (2018).
80. M. A. Chase, S. Stankowski, M. A. Streisfeld, Genomewide variation provides insight into evolutionary relationships in a monkeyflower species complex (*Mimulus* sect. *Diplacus*). *Am. J. Bot.* **104**, 1510-1521 (2017).
81. M. S. Appelhans, C. Paetzold, K. R. Wood, W. L. Wagner, RADseq resolves the phylogeny of Hawaiian *Myrsine* (Primulaceae) and provides evidence for hybridization. *JSE* **58**, 823-840 (2020).
82. L. Liu *et al.*, Phylogeny of *Morella rubra* and its relatives (Myricaceae) and genetic resources of chinese bayberry using RAD sequencing. *PLoS One* **10**, e0139840 (2015).
83. B. Murphy *et al.*, A phylogenomic analysis of *Nepenthes* (Nepenthaceae). *Mol. Phylogenet. Evol.* **144**, 106668 (2020).
84. M. Scharmann, A. Wistuba, A. Widmer, Introgression is widespread in the radiation of carnivorous *Nepenthes* pitcher plants. *Mol. Phylogenet. Evol.* **163**, 107214 (2021).
85. W. Zhou *et al.*, Resolving relationships and phylogeographic history of the *Nyssa sylvatica* complex using data from RAD-seq and species distribution modeling. *Mol. Phylogenet. Evol.* **126**, 1-16 (2018).
86. R. M. Bateman, G. Sramko, O. Paun, Integrating restriction site-associated DNA sequencing (RAD-seq) with morphological cladistic analysis clarifies evolutionary relationships among major species groups of bee orchids. *Ann. Bot.***121**, 85-105 (2018).
87. S. Cozzolino, G. Scopece, L. Roma, P. M. Schlüter, Different filtering strategies of genotyping-by-sequencing data provide complementary resolutions of species boundaries and relationships in a clade of sexually deceptive orchids. *JSE* **58**, 133-144 (2019).
88. L. A. Ripma, M. G. Simpson, K. Hasenstab-Lehman, Geneious! Simplified genome skimming methods for phylogenetic systematic studies: A case study in *Oreocarya* (Boraginaceae). *Applications in Plant Sciences* **2** (2014).
89. H. Kong *et al.*, Phylogenomic and macroevolutionary evidence for an explosive radiation of a plant genus in the Miocene. *Syst. Biol.* **71**, 589-609 (2022).
90. R. Schmickl *et al.*, Phylogenetic marker development for target enrichment from transcriptome and genome skim data: the pipeline and its application in southern African *Oxalis* (Oxalidaceae). *Mol. Ecol. Resour.* **16**, 1124-1135 (2016).
91. M. Zhou *et al.*, Resolving complicated relationships of the *Panax bipinnatifidus* complex in southwestern China by RAD-seq data. *Mol. Phylogenet. Evol.* **149**, 106851 (2020).
92. Y. Ji *et al.*, Genome skimming contributes to clarifying species limits in *Paris* section *Axiparis* (Melanthiaceae). *Front. Plant Sci.* **13**, 832034 (2022).
93. S. Acha, A. Linan, J. MacDougal, C. Edwards, The evolutionary history of vines in a neotropical biodiversity hotspot: Phylogenomics and biogeography of a large passion flower clade (*Passiflora* section *Decaloba*). *Mol. Phylogenet. Evol.* **164**, 107260 (2021).
94. J. G. Chery, C. Sass, C. D. Specht, Development of single-copy nuclear intron markers for species-level phylogenetics: Case study with *Paullinieae* (Sapindaceae). *Applications in Plant Sciences* **5** (2017).
95. D. A. Eaton, R. H. Ree, Inferring phylogeny and introgression using RADseq data: an example from flowering plants (*Pedicularis*: Orobanchaceae). *Syst. Biol.* **62**, 689-706 (2013).

96. C. A. Wessinger, C. C. Freeman, M. E. Mort, M. D. Rausher, L. C. Hileman, Multiplexed shotgun genotyping resolves species relationships within the North American genus *Penstemon*. *Am. J. Bot.* **103**, 912-922 (2016).
97. E. A. Tripp, Y. E. Tsai, Y. Zhuang, K. G. Dexter, RADseq dataset with 90% missing data fully resolves recent radiation of *Petalidium* (Acanthaceae) in the ultra-arid deserts of Namibia. *Ecol. Evol.* **7**, 7920-7936 (2017).
98. X. Ding, J. H. Xiao, L. Li, J. G. Conran, J. Li, Congruent species delimitation of two controversial gold-thread nanmu tree species based on morphological and restriction site-associated DNA sequencing data. *JSE* **57**, 234-246 (2018).
99. R. Medina *et al.*, Phylogenomic delineation of *Physcomitrium* (Bryophyta: Funariaceae) based on targeted sequencing of nuclear exons and their flanking regions rejects the retention of *Physcomitrella*, Physcomitridium and Aphanorrhegma. *JSE* **57**, 404-417 (2019).
100. S. Escobar, A. J. Helmstetter, R. Montufar, T. L. P. Couvreur, H. Balslev, Phylogenomic relationships and historical biogeography in the South American vegetable ivory palms (*Phytelepheae*). *Mol. Phylogenet. Evol.* **166**, 107314 (2022).
101. S. Feng *et al.*, Trans-lineage polymorphism and nonbifurcating diversification of the genus *Picea*. *New Phytol.* **222**, 576-587 (2019).
102. C. Jackson *et al.*, A genome-wide SNP genotyping resource for tropical pine tree species. *Mol. Ecol. Resour.* **22**, 695-710 (2022).
103. D. S. Gernandt *et al.*, Multi-locus phylogenetics, lineage sorting, and reticulation in *Pinus* subsection *Australes*. *Am. J. Bot.* **105**, 711-725 (2018).
104. U. Swenson, J. C. Havran, J. Munzinger, S. McLoughlin, S. Nylinder, Metapopulation vicariance, age of island taxa and dispersal: a case study using the pacific plant genus *Planchonella* (Sapotaceae). *Syst. Biol.* **68**, 1020-1033 (2019).
105. G. Hassemer *et al.*, The application of high-throughput sequencing for taxonomy: The case of *Plantago* subg. *Plantago* (Plantaginaceae). *Mol. Phylogenet. Evol.* **138**, 156-173 (2019).
106. E. Wettewa, L. E. Wallace, Molecular phylogeny and ancestral biogeographic reconstruction of *Platanthera* subgenus *Limnorchis* (Orchidaceae) using target capture methods. *Mol. Phylogenet. Evol.* **157**, 107070 (2021).
107. J. P. Rose, C. A. P. Toledo, E. M. Lemmon, A. R. Lemmon, K. J. Sytsma, Out of sight, out of mind: Widespread nuclear and plastid-nuclear discordance in the flowering plant genus *Polemonium* (Polemoniaceae) suggests widespread historical gene flow despite limited nuclear signal. *Syst. Biol.* **70**, 162-180 (2021).
108. M. Wang *et al.*, Phylogenomics of the genus *Populus* reveals extensive interspecific gene flow and balancing selection. *New Phytol.* **225**, 1370-1382 (2020).
109. Y. Wang *et al.*, Phylogenomics and biogeography of *Populus* based on comprehensive sampling reveal deep-level relationships and multiple intercontinental dispersals. *Front. Plant Sci.* **13**, 813177 (2022).
110. F. C. Boucher, G. Casazza, P. Szovenyi, E. Conti, Sequence capture using RAD probes clarifies phylogenetic relationships and species boundaries in *Primula* sect. *Auricula*. *Mol. Phylogenet. Evol.* **104**, 60-72 (2016).
111. N. Su *et al.*, On the species delimitation of the Maddenia group of *Prunus* (Rosaceae): evidence from plastome and nuclear sequences and morphology. *Front. Plant Sci.* **12**, 743643 (2021).
112. Y. G. Song *et al.*, Phylogeny, taxonomy, and biogeography of *Pterocarya* (Juglandaceae). *Plants* **9** (2020).
113. S. Fitz-Gibbon, A. L. Hipp, K. K. Pham, P. S. Manos, V. L. Sork, Phylogenomic inferences from reference-mapped and *de novo* assembled short-read sequence data using RADseq sequencing of California white oaks (*Quercus* section *Quercus*). *Genome* **60**, 743-755 (2017).

114. J. Cavender-Bares *et al.*, Phylogeny and biogeography of the American live oaks (*Quercus* subsection *Virentes*): a genomic and population genetics approach. *Mol. Ecol.* **24**, 3668-3687 (2015).
115. J. D. McVay, D. Hauser, A. L. Hipp, P. S. Manos, Phylogenomics reveals a complex evolutionary history of lobed-leaf white oaks in western North America. *Genome* **60**, 733-742 (2017).
116. K. K. Pham, A. L. Hipp, P. S. Manos, R. C. Cronn, A time and a place for everything: phylogenetic history and geography as joint predictors of oak plastome phylogeny. *Genome* **60**, 720-732 (2017).
117. K. Debray *et al.*, Unveiling the patterns of reticulated evolutionary processes with phylogenomics: hybridization and polyploidy in the genus *Rosa*. *Syst. Biol.* **71**, 547-569 (2022).
118. J. L. Zhao, B. R. Paudel, X. Q. Yu, J. Zhang, Q. J. Li, Speciation along the elevation gradient: Divergence of *Roscoea* species within the south slope of the Himalayas. *Mol. Phylogenet. Evol.* **164**, 107292 (2021).
119. N. D. Wagner, S. Gramlich, E. Horandl, RAD sequencing resolved phylogenetic relationships in European shrub willows (*Salix* L. subg. *Chamaetia* and subg. *Vetrix*) and revealed multiple evolution of dwarf shrubs. *Ecol. Evol.* **8**, 8243-8255 (2018).
120. L. He, N. D. Wagner, E. Hörandl, Restriction-site associated DNA sequencing data reveal a radiation of willow species (*Salix* L., Salicaceae) in the Hengduan Mountains and adjacent areas. *JSE* **59**, 44-57 (2020).
121. S. I. Lara-Cabrera *et al.*, Phylogenomics of *Salvia* L. subgenus *Calosphace* (Lamiaceae). *Front. Plant Sci.* **12**, 725900 (2021).
122. J. P. Rose *et al.*, Sageinsights into the phylogeny of *Salvia*: Dealing with sources of discordance within and across genomes. *Front. Plant Sci.* **12**, 767478 (2021).
123. J. D. Stephens *et al.*, Resolving phylogenetic relationships of the recently radiated carnivorous plant genus *Sarracenia* using target enrichment. *Mol. Phylogenet. Evol.* **85**, 76-87 (2015).
124. M. Fernandez-Mazuecos *et al.*, The radiation of Darwin's giant daisies in the Galapagos islands. *Curr. Biol.* **30**, 4989-4998 e4987 (2020).
125. J. Heckenhauer, R. Samuel, P. S. Ashton, K. Abu Salim, O. Paun, Phylogenomics resolves evolutionary relationships and provides insights into floral evolution in the tribe *Shoreeae* (Dipterocarpaceae). *Mol. Phylogenet. Evol.* **127**, 1-13 (2018).
126. Y. Li *et al.*, Genomic analyses yield markers for identifying agronomically important genes in potato. *Mol. Plant* **11**, 473-484 (2018).
127. A. M. Duffy *et al.*, Phylogenetic structure in the *Sphagnum recurvum* complex (Bryophyta) in relation to taxonomy and geography. *Am. J. Bot.* **107**, 1283-1295 (2020).
128. H. Y. Lin *et al.*, Phylogenomic conflict resulting from ancient introgression following species diversification in *Stewartia* s.l. (Theaceae). *Mol. Phylogenet. Evol.* **135**, 1-11 (2019).
129. H.-T. Qin *et al.*, Multiple paternally inherited chloroplast capture events associated with *Taxus* speciation in the Hengduan Mountains. *Biorxiv* 10.22541/au.166546663.37338050/v1 (2022).
130. J. R. Jantzen *et al.*, A two-tier bioinformatic pipeline to develop probes for target capture of nuclear loci with applications in Melastomataceae. *Applications in Plant Sciences* **8**, e11345 (2020).
131. M. E. Mort *et al.*, Multiplexed-shotgun-genotyping data resolve phylogeny within a very recently derived insular lineage. *Am. J. Bot.* **102**, 634-641 (2015).
132. E. Leveille-Bourret, B. H. Chen, M. E. Garon-Labrecque, B. A. Ford, J. R. Starr, RAD sequencing resolves the phylogeny, taxonomy and biogeography of *Trichophoreae* despite a recent rapid radiation (Cyperaceae). *Mol. Phylogenet. Evol.* **145**, 106727 (2020).

133. B. M. Anderson, K. R. Thiele, S. L. Krauss, M. D. Barrett, Genotyping-by-Sequencing in a species complex of Australian hummock grasses (*Triodia*): methodological insights and phylogenetic resolution. *PLoS One* **12**, e0171053 (2017).
134. D. Y. Hyun *et al.*, Genotyping-by-Sequencing derived single nucleotide polymorphisms provide the first well-resolved phylogeny for the genus *Triticum* (Poaceae). *Front. Plant Sci.* **11**, 688 (2020).
135. Y. Y. Feng *et al.*, Phylotranscriptomics reveals the complex evolutionary and biogeographic history of the genus *Tsuga* with an East Asian-North American disjunct distribution. *Mol. Phylogenet. Evol.* **157**, 107066 (2021).
136. A. T. Whittemore *et al.*, Phylogeny, biogeography, and classification of the Elms (*Ulmus*). *Systematic Botany* **46**, 711-727 (2021).
137. S. Park *et al.*, Cyclotide evolution: insights from the analyses of their precursor sequences, structures and distribution in violets (*Viola*). *Front. Plant Sci.* **8**, 2058 (2017).
138. L. L. Klein *et al.*, High-throughput sequencing data clarify evolutionary relationships among North American Vitis species and improve identification in USDA *Vitis* germplasm collections. *Am. J. Bot.* **105**, 215-226 (2018).
139. Z. Y. Ma, J. Wen, J. P. Tian, L. L. Gui, X. Q. Liu, Testing morphological trait evolution and assessing species delimitations in the grape genus using a phylogenomic framework. *Mol. Phylogenet. Evol.* **148**, 106809 (2020).
140. Z. Shen, Y. Feng, M. Möller, K. S. Burgess, H. Qin, J. Yang, Z. Mo, H. Li, D. Li, L. Gao, Genomic DNA barcodes provide novel insights into species delimitation in the complex Camellia sect. Thea (Theaceae). BMC Plant Biol. 25, 570 (2025).
141. Y. Ding, C. Metherell, W. Huang, P. M. Hollingsworth, A. D. Twyford, Genome-wide differentiation by geography not species in taxonomically complex eyebrights (Euphrasia). Evolution 79, 483–492 (2025)
