# Supplementary material for: DNA-based identification of plants and the genomic nature of plant species differences
Source: Commun Biol. 2026 Mar 26;9:673. doi: 10.1038/s42003-026-09858-7 (PMC13183946; doi:10.1038/s42003-026-09858-7)
Supplement: Supplementary file 3 — Description of Additional Supplementary Files [file 42003_2026_9858_MOESM3_ESM.pdf]

Description of Additional Supplementary Files:

File name: Supplementary Data 1

Description: Monophyletic Ratio of 151 datasets

File name: Supplementary Data 2

Description: P-values of Welsh's t-test between the monophyly ratio and growth form or sequencing approach

File name: Supplementary Data 3

Description: 27 genera representing 20 plant families with sequence data and metadata available

File name: Supplementary Data 4

Description: Statistics and basic attributes of each species (27 genera)

File name: Supplementary Data 5

Description: Number of SSSNPs in comparison between original and randomised labels

File name: Supplementary Data 6

Description: Number of species told apart by full dataset and whether to keep the dataset in down-sampling. NO means the dataset is excluded from the analysis, YES means included in this analysis. (23 genera)

File name: Supplementary Data 7

Description: The number of species discriminated in rank order of the number of SNPs randomly selected

File name: Supplementary Data 8

Description: Six individual datasets analysed to assess the performance of individual loci for species discrimination

File name: Supplementary Data 9

Description: Single gene diagnosability for *Geonoma*

File name: Supplementary Data 10

Description: Correlation between nucleotide diversity and density of SSSNPs, and nucleotide diversity and number of species that are monophyletic

File name: Supplementary Data 11

Description: Dataset source and contacting information for the owners

File name: Supplementary Data 12

Description: Comparisons between the resolution of nuclear data verses plastome/ITS/other barcodes from a selection of papers.

File name: Supplementary Data 13

Description: A list of datasets used in the meta-analysis. The full data can be downloaded at Zenodo DOI: 10.5281/zenodo.17603347
