# Supplementary material for: DNA-based identification of plants and the genomic nature of plant species differences
Source: Commun Biol. 2026 Mar 26;9:673. doi: 10.1038/s42003-026-09858-7 (PMC13183946; doi:10.1038/s42003-026-09858-7)
Supplement: Supplementary file 5 — Summary [file 42003_2026_9858_MOESM5_ESM.pdf]

nature portfolio

Corresponding author(s): Wu Huang

Last updated by author(s): Nov 2, 2025

Reporting Summary

Nature Portfolio wishes to improve the reproducibility of the work that we publish. This form provides structure for consistency and transparency in reporting. For further information on Nature Portfolio policies, see our Editorial Policies and the Editorial Policy Checklist.

Statistics

For all statistical analyses, confirm that the following items are present in the figure legend, table legend, main text, or Methods section.

n/a	Confirmed	
☐	☒	The exact sample size (*n*) for each experimental group/condition, given as a discrete number and unit of measurement
☐	☒	A statement on whether measurements were taken from distinct samples or whether the same sample was measured repeatedly
☐	☒	The statistical test(s) used AND whether they are one- or two-sided<br>*Only common tests should be described solely by name; describe more complex techniques in the Methods section.*
☒	☐	A description of all covariates tested
☐	☒	A description of any assumptions or corrections, such as tests of normality and adjustment for multiple comparisons
☐	☒	A full description of the statistical parameters including central tendency (e.g. means) or other basic estimates (e.g. regression coefficient) AND variation (e.g. standard deviation) or associated estimates of uncertainty (e.g. confidence intervals)
☐	☒	For null hypothesis testing, the test statistic (e.g. *F*, *t*, *r*) with confidence intervals, effect sizes, degrees of freedom and *P* value noted<br>*Give P values as exact values whenever suitable.*
☒	☐	For Bayesian analysis, information on the choice of priors and Markov chain Monte Carlo settings
☒	☐	For hierarchical and complex designs, identification of the appropriate level for tests and full reporting of outcomes
☒	☐	Estimates of effect sizes (e.g. Cohen's *d*, Pearson's *r*), indicating how they were calculated

Our web collection on statistics for biologists contains articles on many of the points above.

Software and code

Policy information about availability of computer code

Data collection	In the main text following line 465 (currently 466), we have provided a link to our detailed protocol outlining the full inclusion criteria and study identification process: http://dx.doi.org/10.17504/protocols.io.kxygx3z9og8j/v1. This protocol describes the search strategy, screening steps, and criteria used to identify eligible studies. We have clarified this in the manuscript to ensure the process is more immediately transparent to readers.<br><br>In a nutshell, we used ambiguous matching patterns to search in the Web of Science and University of Edinburgh literature search engines for journal publications for studies that sequenced multiple loci from the nuclear genome and which sampled multiple individuals of multiple congeneric species. Details are described in section 1.1 in the protocol. We then filtered the publications by criteria described in section 1.2 in the protocol.
Data analysis	All code used in this study is available at https://github.com/Hazelhuangup/NucBarcoder.<br>The repository includes data processing scripts, analysis workflows, and documentation to reproduce the results presented in this manuscript.

For manuscripts utilizing custom algorithms or software that are central to the research but not yet described in published literature, software must be made available to editors and reviewers. We strongly encourage code deposition in a community repository (e.g. GitHub). See the Nature Portfolio guidelines for submitting code & software for further information.

Data

Policy information about availability of data

All manuscripts must include a data availability statement. This statement should provide the following information, where applicable:

- Accession codes, unique identifiers, or web links for publicly available datasets
- A description of any restrictions on data availability
- For clinical datasets or third party data, please ensure that the statement adheres to our policy

The alignment files, phylogenies, and metadata used in this study can be found in Dryad DOI: 10.5061/dryad.k6djh9wh0. The repositories for raw sequences of the published studies are specified in the cited publications. The list of datasets used in this study can be found in supplementary Table S11.

Research involving human participants, their data, or biological material

Policy information about studies with human participants or human data. See also policy information about sex, gender (identity/presentation), and sexual orientation and race, ethnicity and racism.

Reporting on sex and gender	NA
Reporting on race, ethnicity, or other socially relevant groupings	NA
Population characteristics	NA
Recruitment	NA
Ethics oversight	NA

Note that full information on the approval of the study protocol must also be provided in the manuscript.

Field-specific reporting

Please select the one below that is the best fit for your research. If you are not sure, read the appropriate sections before making your selection.

☒ Life sciences ☐ Behavioural & social sciences ☐ Ecological, evolutionary & environmental sciences

For a reference copy of the document with all sections, see nature.com/documents/nr-reporting-summary-flat.pdf

Life sciences study design

All studies must disclose on these points even when the disclosure is negative.

Sample size	ALL Compile datasets are sample multiple individuals (n>=2) from multiple congeneric species(n>=2) for multiple nuclear markers(n>=3)
Data exclusions	We excluded all datasets where only one individuals have sufficient genomic data. The loci fail a threshold of missing data(for a specific locus, target species must have at least 2 individuals with suffcient data, and for a specific locus, alleles in at least 2 other groups are not missing) are excluded.
Replication	All code and raw data are provided to ensure the repeatability of the analysis.
Randomization	No experimental treatment allocation was performed. Randomisation occurred during computational analyses, in which SNPs and loci were repeatedly and randomly subsampled to assess species discrimination performance at different data volumes (50 replicates per sampling level). Species labels were also randomly permuted to generate null expectations for species-specific SNPs. These procedures ensure unbiased estimation of discriminatory signal.
Blinding	No blinding was performed because the study did not involve experimental interventions, treatment groups, or subjective outcome scoring. All analyses were conducted on publicly available sequence datasets with established species labels. The analyses consisted of automated computational workflows (e.g., SNP calling, sequence alignment, phylogenetic inference), which do not involve investigator judgement that could be influenced by group identity. Therefore, blinding was not relevant to this study.

Reporting for specific materials, systems and methods

We require information from authors about some types of materials, experimental systems and methods used in many studies. Here, indicate whether each material, system or method listed is relevant to your study. If you are not sure if a list item applies to your research, read the appropriate section before selecting a response.

Materials & experimental systems

n/a	Involved in the study	
☒	☐	Antibodies
☒	☐	Eukaryotic cell lines
☒	☐	Palaeontology and archaeology
☒	☐	Animals and other organisms
☒	☐	Clinical data
☒	☐	Dual use research of concern
☐	☒	Plants

Methods

n/a	Involved in the study	
☒	☐	ChIP-seq
☒	☐	Flow cytometry
☒	☐	MRI-based neuroimaging

Dual use research of concern

Policy information about dual use research of concern

Hazards

Could the accidental, deliberate or reckless misuse of agents or technologies generated in the work, or the application of information presented in the manuscript, pose a threat to:

No	Yes	
☒	☐	Public health
☒	☐	National security
☒	☐	Crops and/or livestock
☒	☐	Ecosystems
☒	☐	Any other significant area

Experiments of concern

Does the work involve any of these experiments of concern:

No	Yes	
☒	☐	Demonstrate how to render a vaccine ineffective
☒	☐	Confer resistance to therapeutically useful antibiotics or antiviral agents
☒	☐	Enhance the virulence of a pathogen or render a nonpathogen virulent
☒	☐	Increase transmissibility of a pathogen
☒	☐	Alter the host range of a pathogen
☒	☐	Enable evasion of diagnostic/detection modalities
☒	☐	Enable the weaponization of a biological agent or toxin
☒	☐	Any other potentially harmful combination of experiments and agents

Plants

Seed stocks	All plant material analysed in this study originated from previously published genomic datasets and herbarium specimens as documented in the original publications; no new seed stocks or field collections were generated or acquired.
Novel plant genotypes	No new plant genotypes were generated. All analyses were performed on previously published sequence data.
Authentication	This study did not involve seed stocks or the generation of novel plant genotypes; therefore, authentication procedures and mutation effect analyses were not applicable.
